# Implementation determinants and outcomes in the Philadelphia TelePrEP Program: a mixed methods study of client and staff perspectives

**DOI:** 10.1186/s43058-026-01009-7

**Published:** 2026-06-30

**Authors:** Stephen Bonett, Anna Sweeney, Naomi Correa, Qian Li, Brian Hernandez, Dovie L. Watson, Javontae Williams, Kathleen A. Brady, Denise Gaither-Hardy, Aviva Joffe, Hussein Safa

**Affiliations:** 1 University of Pennsylvania School of Nursing, Philadelphia, USA; 2 University of Pennsylvania, Perelman School of Medicine, Philadelphia, USA; 3 Jefferson Einstein Philadelphia Hospital, Philadelphia, USA; 4 Philadelphia Department of Public Health, Division of HIV Health, Philadelphia, USA; 5 Psychology Department, Lincoln University, Lower Oxford Township, USA

**Keywords:** HIV, Pre-exposure prophylaxis (PrEP), Telehealth, Mixed-methods, Acceptability, Implementation science

## Abstract

**Background:**

HIV pre-exposure prophylaxis (PrEP) is an effective HIV prevention tool, yet uptake and retention among populations vulnerable to HIV acquisition remain below target levels. Telehealth has emerged as a promising strategy to overcome barriers to accessing PrEP. However, significant gaps remain in our understanding of the implementation of telehealth programs for PrEP. This study examines implementation barriers and facilitators in the Philadelphia TelePrEP Program (PTP), assesses program acceptability, usability and satisfaction, and investigates associations between client characteristics and implementation outcomes.

**Methods:**

We conducted a mixed-methods evaluation from October 2023 to February 2025 of the PTP using web-based surveys with 113 participants and in-depth interviews with 5 staff members. We assessed implementation outcomes (i.e., program acceptability, usability, and satisfaction) and explored implementation determinants through qualitative analysis guided by the Consolidated Framework for Implementation Research. We used linear regression to examine associations between sociodemographic and clinical characteristics of participants and implementation outcomes.

**Results:**

Survey participants were predominantly cisgender men with diverse racial/ethnic backgrounds. Participants reported high levels of acceptability, usability, and satisfaction with the PTP, though those with less than full-time employment reported significantly lower acceptability and usability compared to those with full-time employment. Mixed-methods analysis identified patient navigators as a central facilitator of program success, with both staff and clients emphasizing their role in creating a supportive, stigma-free care environment. Key implementation barriers included billing complexity, platform integration challenges, and privacy concerns. Although designed as a fully remote program, substantial demand for in-person services (particularly for injectable PrEP) emerged as a central implementation theme, underscoring the need for flexible hybrid service models. Staff also identified gaps in reaching priority populations, including Black and Latine communities, despite the program’s broad digital outreach efforts.

**Conclusions:**

TelePrEP programs can achieve high user satisfaction when supported by dedicated navigation services and flexible care options. However, realizing the full potential of this model may require additional strategies to enhance program reach, streamline multi-organizational coordination, and support hybrid service models that accommodate diverse patient preferences and socioeconomic circumstances.

**Supplementary Information:**

The online version contains supplementary material available at https://doi.org/10.1186/s43058-026-01009-7.

Contributions to the literature

- Most telePrEP implementation research focuses on either clinical outcomes or patient satisfaction in isolation, with limited understanding of how organizational factors, staff experiences, and patient perspectives interact to enable or hinder equitable program delivery.
- This mixed-methods study reveals that telePrEP programs can achieve high satisfaction when supported by dedicated patient navigation.
- Although designed as a fully remote program, our study reveals substantial demand for hybrid services, particularly for injectable PrEP. This suggests that successful telePrEP implementation requires flexible service models and in-person options.
- These findings expand our understanding of how to scale and sustain telehealth-based HIV prevention programs.

## Background

Since the US Food and Drug Administration’s approval of tenofovir disoproxil and emtricitabine for HIV pre-exposure prophylaxis (PrEP) in 2012, data have demonstrated its effectiveness at preventing HIV acquisition [1, 2] and reducing new HIV diagnoses on a population level [3, 4]. This effectiveness has been stunted, however, by barriers impeding its initiation and sustained use among populations vulnerable to HIV acquisition [5]. 

Limited access to sexual health services has hindered widespread PrEP uptake. Many individuals do not perceive themselves to be vulnerable to acquiring HIV, despite behavior and sexual network factors elevating vulnerability, which can diminish interest in PrEP [6]. Moreover, stigma surrounding sexual health and HIV discourages conversations about PrEP with healthcare providers [7, 8]. Additional challenges include medical mistrust due to experiences of bias in healthcare settings, financial constraints, and structural barriers such as transportation difficulties and lack of social support [7, 9]. 

Telehealth offers a promising strategy to overcome these barriers by improving accessibility, convenience, and privacy in PrEP care. Telecommunication technology has already been used widely to facilitate patient education and offer adherence support [10, 11], and more recently for comprehensive remote PrEP services [12]. These telehealth models for PrEP care (i.e. telePrEP) can improve access, convenience, and adherence, especially for people living far from clinics, facing transportation or scheduling constraints, or having privacy concerns that make in-person care less appealing [13]. 

TelePrEP has been piloted in various settings, demonstrating promising outcomes [14]. The PrEPTECH trial showed six times higher odds of PrEP initiation with telehealth support versus standard online resources, with a 75% initiation rate [15]. The Iowa TelePrEP program achieved an 81% PrEP initiation rate among participants who completed an initial visit and 56% retention rate at 6 months [16, 17]. Where the usability and acceptability of these services have been studied, they have generally been found to be relatively high [18, 19]. 

However, telePrEP implementation and effectiveness depend on factors influencing patient acceptability and engagement, including technological access, healthcare coverage, sociodemographic characteristics, and psychosocial experiences [10]. Some clients report challenges related to privacy, technology navigation, and impersonal provider interactions [20]. Across healthcare contexts, sociodemographic factors, healthcare access, and patient-provider communication influence satisfaction with telehealth [21]. This suggests that acceptability of telePrEP services may vary significantly across different populations, underscoring the need for research on program optimization and equity.

The Philadelphia TelePrEP Program (PTP) was designed to expand PrEP access by integrating telemedicine into the Philadelphia Department of Public Health’s (PDPH) sexual health platform, PhillyKeepOnLoving.com. The program offers flexible, online PrEP services, including self-scheduling, navigator support, options for lab testing (via home specimen self-collection kits or in-person at either the clinic at Jefferson Einstein Philadelphia Hospital or through lab service providers such as Labcorp or Quest), and virtual visits for initiation and follow up. Significant gaps remain in our understanding of telePrEP implementation from multiple stakeholder perspectives. Most existing telePrEP research has focused on clinical outcomes such as PrEP initiation and retention, or has examined patient satisfaction in isolation, without integrating patient and provider perspectives on acceptability. Additionally, there is limited research applying implementation science frameworks to understand the organizational and contextual factors shaping telePrEP program delivery. This study addresses these gaps by applying an implementation determinants framework that integrates both staff and client perspectives as part of a convergent mixed-methods program evaluation. Specifically, we examine implementation barriers and facilitators in the PTP, describe program acceptability, usability and satisfaction, and assess associations between client characteristics and implementation outcomes.

## Methods

### Study design

We conducted a mixed methods study using a convergent parallel design, collecting and analyzing quantitative and qualitative data independently before merging findings to provide complementary perspectives on implementation barriers, facilitators, and outcomes, following guidelines for reporting mixed methods research (Supplemental file 1) [22]. Qualitative data came from in-depth interviews with staff at the PTP conducted August-September 2024. Program clients participated in web-based surveys, with baseline data collection occurring between October 2023 and February 2025, and follow-up surveys conducted 6 and 12 months after baseline.

### Study setting

The study took place in the PTP, a telehealth program for PrEP service delivery developed by the PDPH and implemented by a partner healthcare organization, Jefferson Einstein Philadelphia Hospital (JEPH). JEPH operates the telePrEP clinic in connection with their Immunodeficiency Clinic, a Ryan White funded clinic which provides comprehensive health and social support services to individuals living with HIV, as well as their Pride Clinic, dedicated to serving LGBTQ+ community members. Prospective clients are primarily recruited through digital advertising campaigns across social media and search engine platforms, which direct them to the health department’s sexual wellness website where they can chat with a navigator or self-schedule an appointment. Clients are eligible to enroll if they are Philadelphia residents aged 16 or older. Regardless of insurance status, all eligible clients can access the program; navigators assist uninsured clients with Medicaid applications where eligible, with remaining costs covered through grant funding and 340B cost-savings revenue. The program utilizes a team-based approach including physicians, nurses, and patient navigators who facilitate insurance verification, laboratory coordination, and medication access. Prior to their initial telehealth visit, clients complete laboratory testing either at a local commercial laboratory, at the JEPH clinic, or through mailed at-home specimen self-collection kits processed by a contracted laboratory. Follow-up visits conducted at 3-month intervals for laboratory monitoring and 6-month intervals for telehealth consultations.

### Interviews with program staff

To explore staff perspectives on program implementation, program staff were recruited by total population sampling, with email invitations sent to all staff members directly involved in the telePrEP program (*n* = 8). Staff roles recruited included program administrators, PrEP providers, nurses, PrEP navigators, and support staff. A trained research project manager conducted interviews via video conference. We used a semi-structured interview guide grounded in the Consolidated Framework for Implementation Research (CFIR) (Supplemental File 1) [23]. The guide consisted of open-ended questions written to gain insight into implementation determinants across several key CFIR domains (i.e., innovation, inner setting, outer setting, and individuals). In-depth interviews also included questions to explore elements of the patient-provider encounter and societal context that might shape the implementation of this program [24]. Interviews lasted 25 to 45 min. Audio recordings of interviews were transcribed verbatim and then deidentified for analysis.

### Client survey

To assess client perspectives on program acceptability, we conducted a web-based baseline client survey that recruited telePrEP clients who had successfully initiated contact with program staff, by text, phone, or using the program’s appointment self-scheduler. Clients were eligible after first contact, regardless of subsequent engagement. By including clients across a continuum of program engagement, we gained insight into the reasons potential clients were not engaging further and identified potential barriers in the enrollment process. Eligible clients received invitations via text or email with survey links. Clients who successfully completed the baseline survey were invited to complete follow-up surveys at six months and 12 months after the initial completion date. All quantitative analyses reported here are based on the baseline survey, while the qualitative analysis of free-response data was drawn from both the baseline and follow-up surveys.

#### Measures

##### Usability

Usability of the telePrEP platform was assessed using an adapted version of the two-item Usability Metric for User Experience – LITE scale (“The Philadelphia TelePrEP Program’s features meet my need” and “The Philadelphia TelePrEP Program is easy to use”) [25]. Participants rated their agreement with each statement on a 5-point Likert scale (1="Strongly disagree” to 5="Strongly agree”). A usability score was calculated by averaging responses to these two items, with higher scores indicating greater perceived usability (Cronbach’s alpha = 0.79).

##### Acceptability

Program acceptability was assessed using responses to five items rated on a 5-point Likert scale (1="Strongly disagree” to 5="Strongly agree”). Adapted from previous telePrEP studies, these items assess participants’ perceptions of the advantages of telePrEP compared to traditional service delivery, privacy concerns, trust in the program, and willingness to recommend the service to others (“TelePrEP is a better way to get PrEP, compared to other ways to get PrEP”; “TelePrEP is fast and convenient, compared to other ways of getting PrEP”; “TelePrEP is private and confidential”; “I trust the telePrEP program”; and “I would recommend telePrEP to someone else who might be interested in PrEP.“) [18, 19, 26]. We calculated an acceptability score by averaging responses across all five items, with higher scores indicating greater acceptability (Cronbach’s alpha = 0.82).

##### Satisfaction

Satisfaction with various components of the telePrEP program was measured by asking participants to report which of nine possible program components they engaged with (e.g. “Connecting with a PrEP navigator through chat, text, phone, or video call to discuss PrEP”; “Collecting blood and other lab samples (urine and swabs) using the mailed self-collection kit”; “Connecting with a medical provider in a video appointment and discussing PrEP”), and then rating their experience with each component as “Poor,” “Fair,” “Good,” or “Excellent.” We calculated a standardized satisfaction score by assigning numerical values to these ratings (Poor = 0, Fair = 1, Good = 2, Excellent = 3) and dividing by the total possible score based on the number components a participant had engaged with. This produced a score ranging from 0 to 1, with higher scores indicating greater satisfaction with program components they engaged with.

##### Program status continuum

Participants were categorized into a four-stage program continuum based on their self-reported engagement with program components: (1) “Enrolled in Program” - completed initial enrollment through navigator interaction or self-scheduling; (2) “In Progress” - completed either laboratory testing (via mailed kit or facility visit) or a video appointment with a medical provider, but not both; (3) “Prescription Eligible” - completed both laboratory testing and a medical provider visit, making them eligible for PrEP prescription; and (4) “Received PrEP” - obtained medication through mail or pharmacy pickup.

##### Demographic characteristics

Age was measured as self-reported age in years. Gender identity was collapsed into three categories: “Cisgender man”, " Cisgender woman”, or “Trans/Nonbinary.” Race/ethnicity was derived from questions about Hispanic/Latine ethnicity and racial identity and categorized as " Hispanic/Latine,” “Non-Hispanic White,” “Non-Hispanic Black,” or “Another Race or Multiracial.” Sexual orientation was categorized as “LGBQ” or “Heterosexual.” Education level was measured as the highest level completed and collapsed into three categories: “High School,” “Associate’s/Bachelor’s degree,” or “Graduate/Professional degree.” Employment status was collapsed into a binary variable indicating “Full-time Employment” or “Less than Full-time Employment.” Primary care access was assessed by asking participants if they had a primary care provider and categorized as “Yes” or “No/Don’t know.”

##### Comfort with video calling technology

We assessed participants’ comfort with using video calling technology by asking “How easy is it for you to use a computer or mobile device to make video calls (for example, using FaceTime, Zoom, Skype, or other platforms)?”, with response options rated on a 5-point scale from “Extremely hard” to “Extremely easy”, with higher scores indicating greater comfort with video calls.

##### Sexual health

Prior PrEP use was assessed by asking whether participants had ever taken PrEP before engaging with the program and was categorized as “Yes” or “No.” Recent STI diagnosis was self-reported for the past 12 months, assessed through a two-part question that specified common STIs (gonorrhea, chlamydia, syphilis, trichomoniasis) and timing of most recent diagnosis.

##### Free response questions

In addition to structured closed-ended questions, the survey also included several free response questions. The baseline and follow-up surveys asked participants, “What suggestions do you have for how to make the Philadelphia TelePrEP Program work better for you?”, while the follow-up surveys asked open-ended questions about each particular component of the program, following the satisfaction questions, eliciting detailed responses about their ratings of different program components.

### Data analysis

#### Quantitative analysis

We generated descriptive statistics for all demographic characteristics and measures of program acceptability, satisfaction, and usability. To explore the baseline associations between sociodemographic and clinical characteristics of participants and acceptability, usability, and satisfaction with the program, we conducted three linear regression models, one for each outcome. The models included age, race/ethnicity, gender, education level, prior PrEP use, technology comfort, program stage, access to primary care, employment status, and recent STI diagnosis as predictors. We examined variance inflation factors to detect multicollinearity and inspected model diagnostic plots to assess key modeling assumptions. Diagnostic plots revealed modest departures from normality in the residuals of the acceptability and usability models, consistent with the right-skewed, bounded distributions of these outcomes. Sensitivity analyses using logistic regression with dichotomized outcomes (≥ 4.5 for acceptability and usability; ≥ 0.90 for satisfaction) produced substantively identical findings, supporting the robustness of the linear regression results reported here.

Two participants had missing data for one or more analytic variables. Given this small proportion of missingness, missing data were handled using complete case analysis. All statistical tests were two-sided with significance set at *p* < 0.05. All analyses were conducted using R version 4.3.1 (R Foundation for Statistical Computing, Vienna, Austria).

#### Qualitative analysis

##### Interview

We utilized a rapid qualitative approach to analyze the interview transcripts [27, 28]. We developed a summary sheet that contained a heading for each CFIR construct addressed in the semi-structured interview guide. Each interview was deductively analyzed by two of three authors (AS, QL, and SB), who recorded detailed notes into a summary sheet. After independent analysis, the analysts met to resolve any discrepancies through discussion. This was then adapted into an analysis matrix organized by the same CFIR constructs [29]. Lastly, one analyst (AS) used the matrix to create a detailed analysis summary which consolidated all notes related to a given construct into determinants, resolved redundancies, and assigned each determinant as a barrier or facilitator. The resulting analysis summary contained key facilitators and barriers identified by interview participants organized by CFIR domain.

##### Survey free-response data

To analyze free responses from the client survey, one author (SB) reviewed all responses and developed a preliminary codebook using an inductive thematic content analysis approach [30]. Each free response was considered as a full quote and coded independently by two authors (AS and QL). After independent analysis, the analysts met to resolve any discrepancies through discussion. The codebook and coding were updated iteratively during this discussion to reach consensus on final coding of all responses.

#### Mixed methods data integration

We created a joint display table of quantitative and qualitative data. Key themes were identified from the qualitative analysis summary. For each theme, relevant qualitative data was taken in the form of quotes from the interview summary sheets and the survey free-response matrix. Relevant quantitative data was taken from the quantitative analysis results. We also summarized the integration of qualitative and quantitative data within each theme, and key takeaways related to the implementation of the PTP.

## Results

### Quantitative results

#### Participant characteristics

A total of 113 individuals responded to the baseline survey (Table 1). The mean age of participants was 30.1 years (SD = 9.2). The sample predominantly identified as cisgender men (*n* = 71, 62.8%), with 30.1% (*n* = 34) identifying as cisgender women and 7.1% (*n* = 8) as transgender or nonbinary individuals. The racial/ethnic composition was diverse, with 32.7% (*n* = 37) identifying as non-Hispanic white, 24.8% (*n* = 28) as Hispanic/Latine, 23.9% (*n* = 27) as non-Hispanic Black, and 18.6% (*n* = 21) as non-Hispanic another race or multiracial. Almost half (*n* = 51, 45.1%) reported less than full-time employment. Less than one-third (*n* = 35, 31.0%) had prior experience using PrEP, and 19.5% (*n* = 22) reported an STI diagnosis in the previous 12 months. Participants generally reported high comfort with technology (mean = 4.79 on a 5-point scale, SD = 0.65).

**Table 1:** Summary of telePrEP program evaluation survey respondent characteristics and implementation outcomes at baseline

Variable	Summary (*n* = 113)
Age, mean (SD)	30.12 (9.19)
Gender, n (%)	
Cisgender Man	71 (62.8)
Transgender/Nonbinary	8 ( 7.1)
Cisgender Woman	34 (30.1)
Race/Ethnicity, n (%)	
Hispanic/Latine	28 (24.8)
Non-Hispanic Black	27 (23.9)
Non-Hispanic White	37 (32.7)
Non-Hispanic Another Race/Multiracial	21 (18.6)
Education Level, n (%)	
High School	34 (30.4)
Associate’s/Bachelor’s degree	55 (49.1)
Graduate/Professional degree	23 (20.5)
Employment: Less than full-time, n (%)	51 (45.1)
Has primary care provider, n (%)	73 (64.6)
Prior PrEP Use, n (%)	35 (31.0)
Comfort with technology (scale 1–5), mean (SD)	4.79 (0.65)
STI diagnosis in past 12 months = 1, n (%)	22 (19.5)
Program Status Continuum, n (%)	
Enrolled in program	33 (29.2)
In Progress (Labs OR Visit Complete)	28 (24.8)
Prescription Eligible (Labs + Visit Complete)	12 (10.6)
Received PrEP	40 (35.4)

Survey respondents were similar to the full program population (*n* = 594) but overrepresented cisgender women (30.1% vs. 10%) and Hispanic/Latine individuals (24.8% vs. 5%). The full program population comprised 36% white, 26% Black, 5% Hispanic/Latine, 14% another race/multiracial (19% missing); 84% cisgender men, 10% cisgender women, 5% trans/nonbinary (1% missing).

#### Program engagement and user experience

Participants were distributed across program stages: 29.2% (*n* = 33) had enrolled but not yet completed laboratory testing or a provider visit, 24.8% (*n* = 28) had completed either labs or a provider visit (but not both), 10.6% (*n* = 12) had completed both components and were prescription-eligible, and 35.4% (*n* = 40) had received PrEP medications. Overall, participants reported high acceptability (mean = 4.39 out of 5, SD = 0.76), usability (mean = 4.46 out of 5, SD = 0.66), and satisfaction (mean = 0.82 out of 1, SD = 0.19) with the program (Table 2).

**Table 2:** Acceptability, usability, and satisfaction with Philadelphia TelePrEP Program overall and by progression through program components among baseline survey respondents (*n* = 113)

	Acceptability (scale 1–5), mean (SD)	Usability (scale 1–5), mean (SD)	Satisfaction (scale 0–1), mean (SD)
**Overall**	4.39 (0.76)	4.46 (0.66)	0.82 (0.19)
**Program Status Continuum**			
Enrolled in program	4.35 (0.73)	4.23 (0.69)	0.80 (0.22)
In Progress (Labs OR Visit Complete)	4.40 (0.66)	3.98 (0.99)	0.80 (0.19)
Prescription Eligible (Labs + Visit Complete)	4.75 (0.36)	4.79 (0.40)	0.88 (0.12)
Received PrEP	4.50 (0.67)	4.70 (0.50)	0.85 (0.16)

### Factors associated with program acceptability, usability, and satisfaction

Multiple regression analyses revealed several significant associations between participant characteristics and program implementation outcomes (Table 3). The overall models were statistically significant for acceptability (F(11,99) = 2.97, *p* < 0.01, adjusted R² = 0.16) and usability (F(11,99) = 2.53, *p* < 0.01, adjusted R² = 0.13), but not for satisfaction (F(11,99) = 1.36, *p* = 0.20, adjusted R² = 0.03).

**Table 3:** Multivariate regression models for acceptability, usability, and satisfaction in the Philadelphia TelePrEP Program among users at baseline assessment (*n* = 111)

Overall statistics	Acceptability				Usability				Satisfaction			
	F (11,99) = 2.97 *p* = < 0.01; adjusted *R*^2^= 0.16				F (11,99) = 2.53 *p* = < 0.01; adjusted *R*^2^= 0.13				F (11,99) = 1.36 *p* = 0.20; adjusted R^2^= 0.03			
**Variable**	b	SE	95% CI	p	b	SE	95% CI	p	b	SE	95% CI	p
Age	0.01	0.01	[0.00, 0.03]	0.09	0.01	0.01	[-0.01, 0.03]	0.29	0.00	0.00	[0.00, 0.01]	0.55
Gender (ref= Cisgender Man)	--	--	--	--	--	--	--	--	--	--	--	--
Trans/Nonbinary	0.02	0.22	[-0.43, 0.46]	0.94	-0.21	0.27	[-0.75, 0.33]	0.43	-0.14	0.07	[-0.28, 0.00]	0.05
Cisgender Woman	0.15	0.14	[-0.12, 0.43]	0.27	-0.19	0.17	[-0.52, 0.14]	0.26	-0.05	0.04	[-0.13, 0.04]	0.28
Racial/ethnic minority	-0.02	0.13	[-0.27, 0.23]	0.86	0.07	0.15	[-0.23, 0.37]	0.62	0.00	0.04	[-0.08, 0.08]	0.96
Education Level: High school or less	0.01	0.15	[-0.28, 0.31]	0.92	0.11	0.18	[-0.25, 0.47]	0.54	0.01	0.05	[-0.08, 0.10]	0.79
Employment: Less than full-time	-0.38	0.13	[-0.64, -0.13]	< 0.01**	-0.39	0.16	[-0.70, -0.08]	0.01*	-0.06	0.04	[-0.14, 0.02]	0.15
Has primary care provider	0.19	0.12	[-0.05, 0.43]	0.13	-0.08	0.15	[-0.37, 0.21]	0.59	0.02	0.04	[-0.06, 0.09]	0.61
Prior PrEP Use	0.13	0.14	[-0.14, 0.40]	0.35	-0.09	0.16	[-0.41, 0.23]	0.58	-0.03	0.04	[-0.12, 0.05]	0.44
Comfort with technology (1–5)	0.14	0.09	[-0.05, 0.33]	0.14	0.08	0.11	[-0.15, 0.30]	0.50	0.04	0.03	[-0.02, 0.10]	0.15
STI diagnosis in past 12 months	0.36	0.15	[0.06, 0.66]	0.02*	0.07	0.18	[-0.30, 0.43]	0.71	0.07	0.05	[-0.02, 0.17]	0.12
Program Status Continuum (1–4)	0.05	0.05	[-0.06, 0.16]	0.38	0.16	0.06	[0.03, 0.29]	0.02*	0.01	0.02	[-0.02, 0.04]	0.58

* *p*<0.05, ** *p*<0.01

Participants with less than full-time employment reported lower acceptability (b = -0.38, 95% CI [-0.64, -0.13], *p* < 0.01) and lower usability (b = -0.39, 95% CI [-0.70, -0.08], *p* = 0.01) compared to those with full-time employment. Participants who had been diagnosed with an STI in the past 12 months reported higher acceptability of the telePrEP program (b = 0.36, 95% CI [0.06, 0.66], *p* = 0.02). Progression through the program continuum was positively associated with perceived usability (b = 0.16, 95% CI [0.03, 0.29], *p* = 0.02). No significant associations were observed between program implementation outcomes and age, race/ethnicity, education level, having a primary care provider, prior PrEP use, or comfort with technology in the multivariate models. Given the non-significant overall model for satisfaction, we conclude that the included predictors did not collectively explain a significant amount of the variance in satisfaction and consequently; thus, individual coefficients were not interpreted for this model.

### Qualitative results and mixed methods integration

We conducted in-depth interviews with five staff members. Two interviewees were licensed clinical providers (e.g., physicians, advanced practice providers, and nurses) and three interviewees were administrative and support staff (e.g., patient navigators, chat operators, and program managers). Integration of quantitative and qualitative findings revealed convergent patterns in PTP implementation and participant experiences. The joint display table (Table 4) illustrates how staff interviews, participant qualitative feedback, and survey data triangulated around seven themes.

**Table 4:** Joint display table integrating qualitative findings from staff and clients with quantitative findings from the client survey

Key Theme	Staff Qualitative Interviews	Client Qualitative Survey Free Text Responses	Client Quantitative Data from Surveys	Mean (SD)	Integration and Takeaway
Theme 1: Navigators and Other Support Staff (Inner Setting: Available Resources and Communications)	“[To expand] we need more staff for some positions. We have a lot of admin positions, but not a lot of clinical positions. [In-person staff] would help us to facilitate the follow ups with the labs and all that. Also, when we get more patients having another navigator would also help to keep track of the patients that we have.” [2] “We started with one doctor, who was able to precept the residents that were doing the appointments with patients. And now we have two doctors who are doing that. So that opened up time slots and availability. We are offering one Saturday a month of appointments, and then sometimes nighttime appointments to accommodate people’s schedules. And so I think, more providers, means more appointment availability.” [3]	“I loved the convenience of scheduling appointments through text and chat without even needing to call them.”—10 Six-month Follow-up “Connecting with the navigators via text feels very informal and haphazard. I’m often confused about when my next appointment is, who I’ll be seeing, and who I should be in contact with.”—47, Six-month Follow-up	*Strongly Agree = 5* *Strongly Disagree = 1*	*6-Month Follow-Up Data* *(n* *= 55)*	Navigators and support staff are an important factor to the success and growth of the program. A robust support staff with navigators to handle scheduling, insurance navigation, lab work coordination, and billing are key factors for program growth. Additionally, the availability of staff to communicate with clients, provide updates, and answer questions is a key component of client satisfaction. Clients even suggested more communication from staff to anchor the remote process and help guide them through the journey.
			When I reach out to the Philadelphia TelePrEP Program for help, they respond in an appropriate amount of time.	4.28 (1.02)	
			I have a way to contact the program when I need to.	4.22 (0.93)	
			*Excellent = 4* *Poor = 1*	*Baseline Data* *(n* *= 113)*	
			Rate your experience connecting with a PrEP navigator through chat, text, phone, or video call to discuss PrEP	3.59 (0.58)	
Theme 2: Billing and Insurance (Innovation: Complexity)	“[We hear] things like, ‘I’m tired of getting a bill for something that you told me is taken care of’, and we say, ‘No, no, it is, I promise, don’t pay it.’ But they’ll say, ‘It’s going to collections.’ So, we have had very few people choose not to continue with the program because of the mess that that has caused.” [2] “A problem that we’re still working on trying to solve is patient billing. The program is advertised as free, it’s really important to patients that it’s free, it’s important to us that it’s free, but actually making that happen requires adapting to a level of bureaucracy that is difficult to navigate.” [1]	“I’m getting bills for the program even though it’s supposed to be free. My primary insurance is billed and TelePrEP is secondary insurance, yet I continue to get LabCorp bills with outstanding balance. I’ve had multiple communications with the TelePrEP team, and no one can seem to finalize these bills.”—7, 12-Month Follow-up “The first time I went to a LabCorp, I was required to pay for my testing and was unable to be reimbursed for it as I thought I would have been able to.”—103, Six-month Follow-up	*Strongly Agree = 5* *Strongly Disagree = 1*	*6-Month Follow-Up Data* *(n* *= 55)*	Billing is the largest source of patient dissatisfaction with the program. While billing and insurance complications impact any health service organization, this issue does highlight the amount of coordination and integration between institutions (labs, providers, insurers, patient assistant programs, etc.) that is needed to maintain a large telePrEP program.
			I receive appropriate updates from the program about any communications with my insurance company.	3.79 (1.06)	
Theme 3: Platform Integration (Innovation: Design)	“I think the website is definitely confusing because it’s bundled in with other Philly resources and a lot of times we’ll get like appointment schedules for like … HIV exposure or other STI questions about testing or treatment. So I think the website itself, it can be misleading to patients.” [4] “One issue at the beginning was like figuring out the self-scheduling because the website can’t directly interface with our EMR. In an ideal world, patients would be able to just schedule directly on the website, and then we would receive that and their position in the schedule would be held, but there’s just no way to do that. Instead, we have to have links to the website that we use for self-scheduling. Figuring out how to put the links up, we had different departments we were working with at the time, so that got really complicated. But now we’ve streamlined it down to just like one link that works.” [1]	“Going to LabCorp is quick and simple, and I can see the results in the Einstein patient portal. To see the results of the mail-out testing, I have to have a coordinator email them to me. That aspect of the mail-out testing is annoying. It would be nice if the results could just be posted to the patient portal like the LabCorp results are.”—47, Six-month Follow-up “To get results for the chlamydia and gonorrhea tests, I had to call multiple numbers to direct me to the right one- the one on the website wasn’t working. I think updating the *Philly Keep [on] Loving* website would be good. Even better would be sending an email with test results- it’s hard to ask for test results from other people to have safe sex if I don’t have a copy on my end, but I can’t afford testing at labs send digital results.”—95, Baseline	*Excellent = 4* *Poor = 1*	*Baseline Data* *(n* *= 113)*	The Philadelphia TelePrEP Program was developed by the Philadelphia Department of Public Health and implemented by JEPH. Because of this, some aspects of the program, like the landing page from the advertisements, are part of the PDPH network. Patients who self-schedule appointments start on the PDPH website before transitioning to the JEPH patient portal, where those that are not existing JEPH patients must create new accounts and enter demographic and insurance information. Navigators then conduct follow-up PrEP-specific intake calls. This structure required intensive collaboration between PDPH, JEPH, and their IT departments to create a seamless user experience, and means that JEPH staff must sometimes help clients navigate website content they do not control.
			Rate your experience using the self-scheduling tool to schedule an appointment with a medical provider	3.57 (0.58)	
			*Strongly Agree = 5* *Strongly Disagree = 1*	*6-Month Follow-Up Data* *(n* *= 55)*	
			I receive appropriate updates from the program about my lab results.	4.37 (0.86)	
			I receive appropriate updates from the program about when my test kit arrives at the lab after I return it.	4.08 (1.31)	
Theme 4: Demand for Hybrid Service Model (Innovation: Adaptability)	“We were thinking in the beginning- Oh, most of the people want to do like everything remote. They want to be with the kids at home. They want to get the prescription delivered. And I think that’s the whole point when you think about TelePrEP service. But because we’re posted in Einstein, and they already have the option for in-person injectable [PrEP], we were very surprised with the amount of [in-person] patients. Actually, we have more patients on injectable PrEP from the TelePrEP program than the program for the in-person PrEP.” [2] “Something we talk about when we talk about injections is that you have to be able to come between 9 to 5, Monday through Friday, which is our injection hours. I’d love for there to be a different site where people can also go and get their injections.” [5]	“I really wanted to stick with the injectable but switched to the pills. I was only given one clinic open, and it was 30–45 minutes way from my house, upwards to an hour away with traffic. Not to mention, they are only open during my work hours. And I’d usually have to wait 30-45ish minutes when I was there before I was seen. So in total, I was missing up to 3 hours of work and I couldn’t do that.”—21, Six-month Follow-up “A list of potential locations for care would have been helpful if there was an option closer to my home. The [clinic] is not convenient to get to, but it’s manageable. A closer location may have been helpful to receive care.”—26, Baseline	*Excellent = 4* *Poor = 1*	*6-Month Follow-Up Data* *(n* *= 55)*	There is substantial demand from clients for a hybrid model of services, where options are available to receive care in-person. Specifically, the demand for injectable PrEP was higher than anticipated, which prompted expansion of the program to include an additional medical assistant (funded through 340B revenue) to support the one registered nurse who initially handled all in-person appointments.
			Rate your experience receiving Injectable PrEP (e.g. Apretude [Cabotegravir]) in the clinic.	3.42 (1.13)	
			Rate your experience of going to the clinic to have blood and other lab samples collected.	3.50 (0.63)	
Theme 5: Privacy and Information Sharing (Individual: Need)	“A lot of times patients have insurance, but maybe they’re under their family’s plan or partner’s plan, and they don’t want them to know that they’re using their insurance for these services. So their options for getting medication are definitely more limited.” [4] “I think stigma [is a barrier] … I think sometimes people don’t even know what PrEP is, or that it exists. I think oftentimes people stumble into PrEP because of PEP or a close call, or you know, that’s when you’re really the most nervous or worried. That’s when you’re the most likely to take some action. I think there’s stigma associated with any of this.” [5]	“The only problem I’ve had is that it was not made explicitly clear to me prior to starting the program that me taking PrEP would be shared on [the patient-facing electronic health record portal] with the rest of my doctors. It’s too late to take back now, but I wish I’d been told this outright so that I could have weighed the benefits against the risk of discrimination from other doctors and made an informed decision. Instead, I felt disempowered in my own medical care yet again. Please make this clearer to future participants prior to starting the program.”—70, Baseline	*Strongly Agree = 5* *Strongly Disagree = 1*	*Baseline Data* *(n* *= 113)*	Privacy is an important factor for patients as they initiate contact with the program and contemplate enrollment. While those in the program rated it as highly trustworthy, this could miss the perceptions of people who are wary and decide not to initiate contact with the program at all. Flexibility for each client’s journey to accommodate hesitation is a strength of the program and its enrollment success. Participants also raised concerns about unintended disclosure of PrEP use through shared electronic health records, an issue affecting all PrEP care models that underscores the need for transparent informed consent about information sharing.
			TelePrEP is private and confidential.	4.58 (0.82)	
			I trust the telePrEP program.	4.60 (0.75)	
Theme 6: Appropriateness of TelePrEP Model (Innovation: Relative Advantage and Inner Setting: Compatibility)	“Something that I take a lot of pride in, and I think is super important, is that we’re training new doctors not only in providing PrEP, but in being inclusive and accessible. And so that is something that we will feel for years to come. As more doctors feel comfortable with this, it’ll make PrEP access a lot wider because there’s a lot of primaries who won’t do it. And primaries totally can, they are fully capable of doing it, they just need the proper training to do so. The more providers who can give it, the more people will access it.” [1] “We take a very patient centered approach. We tell them what their options are. We tell them why these are the options, and why other things aren’t the options, and then we just let them make a decision.” [5]	“More appointments at night (or even on weekends) would be beneficial for me.”—18, Baseline “The mailing of the kits is fine. I think additional supplies could be included or walk-through of blood collection. There have been times where I bled too much then another when not enough to collect a sample.” —60, Six-month Follow-Up	*Strongly Agree = 5* *Strongly Disagree = 1*	*Baseline Data* *(n* *= 113)*	For the client, the program provides increased flexibility and allows for a patient-centered approach that can be adjusted to meet the needs of the individual, and they rated it highly overall. A few participants had specific suggestions about how to improve the telehealth experience related to scheduling and lab kits. For staff, the telePrEP model was seen as a more flexible platform to both meet patients where they are and improve the availability of resources in the Philadelphia community, including through its contributions to training medical residents in PrEP care.
			TelePrEP is fast and convenient, compared to other ways of getting PrEP	4.27 (1.03)	
			TelePrEP is a better way to get PrEP, compared to other ways to get PrEP	4.13 (1.00)	
			I would recommend telePrEP to someone else who might be interested in PrEP	4.70 (0.65)	
Theme 7: Recruitment and Outreach (Outer Setting: Local Conditions)	“I think my main [goal] is just like getting it to the people who need it the most, and I still feel like the marketing and targeting of people isn’t necessarily capturing that. I’m pretty sure the majority of our patients are still white, gay men. We do have a more diverse representation. But, probably in the 70% range might be white.” [3] “The ads are so extremely influential to the number of patients we receive. Like if the ads get turned off, we get nobody. Our inbox is empty, no one is scheduling, it halts the entire process.” [1]	“Not specifically for me but increasing the ad budget and blanketing locations that cater to those who would most benefit from PrEP.”—2, Six-month Follow-up “Outreach efforts could be stronger possibly. On foot traffic in the city. Setting up pop up locations in places around the city or in areas heavily impacted by HIV transmissions. HIV and PrEP programs to collab more at events as well if not done already.”—60, Six-month Follow-up “More outreach, people don’t know about the program.”—107, Baseline	*How did you hear about TelePrEP?* *(Select all that apply)*	*Baseline Data* *(n* *= 113)* *n(%)*	While the ad campaign was the driving force to reach key populations in the early years of implementing this program, perspectives from clients and staff suggest that new avenues of outreach may be needed to continue to expand reach. These might include print campaigns, community partnerships, or program ambassadors that would promote greater awareness of the telePrEP program across different communities.
			I saw information about it on social media.	43 (25.90%)	
			I saw information about it on Google or another website.	75 (45.18%)	
			I heard about it from a friend.	28 (16.87%)	
			My doctor, nurse, or other medical provider told me about it.	10 (6.02%)	
			Other	10 (6.02%)	

#### Theme 1: Navigators and other support staff (inner setting: available resources and communications)

In interviews with clinical providers, they identified the navigators, chat operators, and registered nurses as key factors in the program’s growth. As the patient load increases, their work behind the scenes allows providers to increase their available appointment slots.

Having [nurses and navigators] who can counsel about STIs and DoxyPEP… can answer questions, can navigate things, can talk to insurance… It makes my job a whole heck of a lot easier because I’m not trying to do everything else. [Staff Member, 5]

In addition to the navigators being key in the expansion of the program, the availability of navigators improves patient satisfaction with the program by providing a compassionate point-of-care.

Being able to text with someone was so helpful, and the warmth, care and compassion that person had was so awesome, because it demonstrated that there would be no judgment from that person. [Client 2, Six-month Follow-Up]

However, the client feedback also revealed a desire for more frequent communication from staff. Clients expressed desire for faster response time, appointment reminders, text reminders to return the home specimen self-collection kit, and updates when the kit has been received.

Connecting with the navigators via text feels very informal and haphazard. I’m often confused about when my next appointment is, who I’ll be seeing, and who I should be in contact with. [Client 47, Six-Month Follow-Up]

#### Theme 2: Billing and insurance (innovation: complexity)

All five staff members identified billing as the primary issue in the rare cases of patient dissatisfaction. Even when telePrEP services are provided without cost-sharing for clients, staff must coordinate billing through multiple entities including various insurance carriers, third-party laboratories (such as those processing home specimen collection kits), patient assistance programs, and different departments within JEPH. This complex coordination creates opportunities for billing errors and delays. Most patients never received bills, as services were covered by insurance or grant funding. However, in some cases (e.g., third-party labs), bills were generated requiring staff coordination to resolve charges. This can create frustration and tension, as one staff member explained:

[Billing] is the biggest issue right now just because it can really influence how patients perceive the program. And we’ve had people leave because of it. [Staff Member, 1]

Miscommunications around billing can affect patients’ trust in and perception of the program, which was reflected in one client’s feedback.

I have had major issues related to finances and this service. I have gotten collections notices which has been scary…I feel lied to and dismissed. [Client 74, Six-month Follow-Up]

#### Theme 3: Platform integration (innovation: design)

Implementation challenges emerged from the integration of multiple institutions and organizations in the program’s structure and remote delivery model. The PTP was developed by PDPH and implemented by JEPH, creating a disconnect where JEPH staff must help clients navigate a website managed by a different organization.

The website hasn’t been that user friendly… A lot of my job is fielding questions about where to get STI resources… there’s got to be something with the messaging that is leading people to believe that they’re making the appointment for [STI testing and treatment]. [Staff Member, 3]

While some connections across platforms resulted in a smooth integration, such as test results from major lab service providers like Labcorp and Quest appearing directly in the patient portal, other lab partnerships lacked electronic integration. Mail-in specimen kits were processed by a laboratory without direct electronic health record connectivity, requiring staff to manually retrieve and share results with clients.

Going to [a local lab] is quick and simple, and I can see the results in the Einstein patient portal. To see the results of the mail-out testing, I have to have a coordinator email them to me. That aspect of the mail-out testing is annoying.—Client 47, Six-month Follow-up.

#### Theme 4: Demand for hybrid service model (innovation: adaptability)

While the telePrEP program was designed to be fully remote, clients who want to use certain clinical services like in-person lab collection or injectable PrEP must come to the in-person clinic, resulting in a hybrid care model (i.e., some elements of care delivered virtually and some elements delivered in person). This hybrid model proved to be a strength of the program, allowing flexibility and choice for clients. Specifically, clients seeking injectable PrEP added to the demand for in-person services. All five staff members interviewed mentioned that the number of patients who opted for injectable PrEP was larger than anticipated.

However, the hybrid model also introduced travel and scheduling as barriers to care; unlike the remote provider consultations, in-person services must be completed during business hours for the clinic. One client highlighted this challenge:

I really wanted to stick with the injectable but switched to the pills. I was only given one clinic option and it was 30–45 min away from my house, upwards to an hour away with traffic. Not to mention, they are only open during my work hours. [Client 21, Six-month Follow-Up]

#### Theme 5: Privacy and information sharing (individual: need)

Despite the potential for increased privacy through the telehealth model, concerns about how clients’ information would be shared, and with whom, remained relevant. In interviews, three of the five staff members cited stigma surrounding HIV and PrEP in the community as a deterrent to enrollment, especially for young adults. One said:

People who are young and are on their parent’s insurance that are afraid that’s going to show up or a mail order kit is going to expose them to their parents. [Staff Member, 3]

Additionally, some clients explicitly expressed concern over the safety of their personal information.

It was not made explicitly clear to me… that me taking PrEP would be shared on the [EMR] with the rest of my doctors… I wish I’d been told this outright so that I could have weighed the benefits against the risk of discrimination from other doctors. [Client 70, Baseline]

#### Theme 6: Appropriateness of TelePrEP model (innovation: relative advantage and inner setting: compatibility)

The telePrEP model provides unique advantages from both the clients and staff perspectives. For the clients, the program provides increased flexibility and allows for a patient-centered approach. For staff, the program allows providers to meet patients where they are. One staff member explained:

Equity, reaching folks who don’t have access to PrEP, bringing these new options for PrEP to a population that otherwise doesn’t get access to things like [injectable PrEP]… to bring these new innovations to a population… is always a big goal. [Staff Member, 5]

Additionally, JEPH operates the PTP in connection with their Immunodeficiency Clinic and their Pride Clinic. This provides a unique opportunity to integrate medical residents into the program. Residents on rotation in the clinic were given the opportunity to conduct telePrEP visits under the supervision of the telePrEP providers.

I think a kind of underrated aspect of the program is the fact that we’re training residents on providing accessible, inclusive, trauma-informed PrEP care and I think that is a ripple effect that is super important. [Staff Member, 1]

Clients appreciate the flexible nature of the program and even state that it helps them prioritize their own health; one client mentioned, “The ease of collecting samples from my own home helped me from further delaying testing” [Client 40, Six-month Follow-Up]. However, the at-home dried-spot blood collection was occasionally perceived to be difficult to complete, with some clients stating the kits would benefit from clearer instructions.

The mailing of the kits is fine. I think additional supplies could be included or walk-through of blood collection. There have been times where I bled too much then another when not enough to collect a sample. [Client 60, Six-month Follow-Up].

#### Theme 7: Recruitment and outreach (outer setting: local conditions)

All five of the staff members interviewed cited the online ad campaign as the main source of new patients. One aim of the recruitment campaign was to engage key underserved populations, specifically Black and Hispanic/Latine communities.

Off hand, I do know that about 70% of our patients are white, predominantly MSM… But if we look at certain groups like Black cis women, trans women, MSM of color like those categories are quite small and not proportional to the population. [Staff Member, 1]

Several staff members felt there are missed opportunities to diversify the recruitment campaign to try to reach these communities. Staff suggested that a print campaign on buses or billboards could expand reach. Additionally, staff members highlighted opportunities to partner with community programs to increase awareness of the PTP. One said:

I think to expand, we’re going to have to partner within these communities. We’re going to have to use real people and real patients who are on PrEP to bring more people into it, so word of mouth. [Staff Member, 5]

Clients echoed this, with one mentioning their perception that the program had minimal presence in-person in the community.

Outreach efforts could be stronger… Setting up pop up locations in places around the city or in areas heavily impacted by HIV transmissions [or at local events]. [Client 60, Six-month Follow-Up]

## Discussion

This mixed-methods evaluation of a telePrEP program assessed implementation barriers and facilitators, program acceptability, usability and satisfaction among users, and associations between client characteristics and implementation outcomes. We found that participants reported consistently high levels of acceptability, usability, and satisfaction. Our mixed-methods analysis identified seven implementation themes that shaped the patient journey through the program. These findings demonstrate that telehealth-based PrEP delivery can achieve high user satisfaction while revealing specific operational considerations essential for successful implementation and scale-up of telePrEP services.

Our findings highlight patient navigators as an essential element of a successful telePrEP program. Both staff interviews and client feedback consistently identified navigators as key facilitators who monitor and guide each patient’s individualized journey through the program while maintaining clear communication about care plans and next steps. Participants reported high satisfaction with staff responsiveness and accessibility, with qualitative feedback emphasizing that the staff helped create a safe, stigma-free environment for accessing PrEP services. This aligns with broader evidence demonstrating the effectiveness of telehealth navigators, where previous research has found that patients who work with navigators have greater appointment attendance and improved care engagement [31, 32]. 

However, analysis revealed tensions in the navigator role: while participants appreciated text-based communication, they expressed confusion about logistics, suggesting opportunities to streamline coordination. The complexity of the navigator role was particularly evident in billing-related challenges, where navigators must bridge gaps between JEPH’s internal systems, PDPH’s platform, and external laboratory partners. While insurance navigation and medical billing challenges are not unique to telehealth settings [33], the telePrEP model’s reliance on partnerships outside the primary clinic creates additional layers of complexity that require dedicated navigator intervention to resolve billing discrepancies and maintain patient trust in the program. To address these challenges, programs should consider developing standardized scripts for navigators to use when discussing insurance and billing expectations, alongside written materials for patients that clearly outline the billing process and timeline.

Privacy emerged as a critical implementation factor that could influence program engagement and retention. While enrolled participants reported high levels of trust and perceived privacy/confidentiality in our survey data, staff interviews revealed some participants had privacy concerns that could create barriers to enrolling in the program. This finding aligns with broader research identifying privacy as a significant concern in telehealth services, given that patients may be attending their visits in shared living spaces or environments where confidentiality cannot be guaranteed [34]. Privacy concerns have been specifically documented in HIV care contexts, where patients who opted out of telehealth services often cited confidentiality worries as a primary reason for preferring in-person care [35, 36]. These concerns are often mirrored by healthcare providers themselves, who express their own worries about protecting patient privacy during telehealth appointments [35, 37]. Previous research has pointed to several strategies for addressing and mitigating privacy-related concerns in HIV telehealth, including proactively asking patients at the start of each visit whether they are in a private space where they can speak freely, providing clear information about privacy protections to all patients, and referring patients with persistent privacy concerns to in-person programs [34, 35]. 

Despite being originally conceived as a fully remote PrEP service model, both staff and client feedback revealed substantial and growing demand for in-person components, particularly for injectable PrEP administration. This demand sometimes exceeded the program’s single-site capacity and highlighted geographic barriers for participants who needed to travel significant distances for in-person services, demonstrating that even telehealth-focused programs require robust hybrid service models to meet diverse patient needs and preferences. The demand for injectable PrEP is likely to intensify in the coming years as implementation of this modality improves and new longer-acting formulations have now entered the market [38]. One of the program’s key strengths lies in its flexibility, allowing patients to choose from multiple in-person and remote options at each stage of the care process. This aligns with broader research emphasizing the importance of expanding patient choices regarding how, when, and where they receive sexual health services to promote access and engagement [35]. 

Our findings point to several equity considerations that highlight the need for expanded and diversified outreach strategies to achieve the program’s goals of serving underserved populations. It is important to note that the high levels of acceptability, usability, and satisfaction reported here reflect the experiences of individuals who successfully engaged with the program; they should not be interpreted as evidence of broad program accessibility, as barriers may exist that prevented individuals from enrolling in the program in the first place. Participants with less than full-time employment reported significantly lower acceptability and usability scores compared to those with full-time employment, suggesting that socioeconomic factors continue to influence telehealth engagement even when services are provided at no cost. This aligns with research identifying digital inequities along socioeconomic lines affecting telehealth access and utilization, including technology access, digital literacy, internet connectivity, and private spaces [34, 39]. Staff interviews confirmed that despite the program’s success in reaching hundreds of participants, representation of Black and Latine communities was disproportionately low compared to HIV incidence rates in these communities locally [40]. Telehealth delivery alone may be insufficient to address long-standing racial disparities in PrEP access. Despite eliminating transportation barriers and offering services at no cost, Black and Latine individuals continued to face barriers to accessing this PrEP care model. This pattern reflects broader research warning that digital health interventions can reproduce and even amplify existing health inequities when they fail to address underlying structural barriers [41, 42]. 

The staff and client perspectives on recruitment highlight both the successes and ongoing challenges of various marketing and advertising strategies. At the time of these interviews, staff identified online advertising as a primary enrollment driver and suggested additional strategies including print campaigns. Recently, PDPH has expanded existing community engagement efforts that go well beyond online marketing, including major sponsorships and active presence at community events, and program data indicate these efforts effectively drive traffic to the PTP. Notably, PDPH has not prioritized print campaigns, as evidence from their internal evaluations have suggested this strategy is not cost-effective and may have limited reach. To optimize recruitment overall and better engage with priority populations (e.g., Black and Latine individuals, cisgender women, transgender individuals, and people who inject drugs), telePrEP programs may benefit from exploring community-based engagement strategies informed by the perspectives of program staff and clients. Potential approaches suggested by our findings include peer ambassador programs that leverage current PrEP users’ networks, partnering with commuanity organizations to provide in-person navigation support, and expanding service hours into evenings and weekends. Future implementation research should evaluate the feasibility and effectiveness of these strategies in diverse telePrEP settings.

This study is subject to several notable limitations. First, the sample may not fully represent the program population or PrEP-eligible individuals. Participants were predominantly cisgender men with high technology comfort, potentially limiting generalizability. While survey respondents were broadly similar to the full program population (*n* = 594), cisgender women (30.1% vs. 10%) and Hispanic/Latine individuals (24.8% vs. 5%) were notably overrepresented among survey respondents. As a result, our regression coefficients may not generalize to the full program population and may capture dynamics more relevant to the overrepresented groups than to the true program population. Second, data collection occurred during the early implementation phase of the PTP, when some implementation challenges were still being addressed. Our findings may therefore reflect early adoption barriers rather than the program’s mature operational state. Third, those who discontinued the program early or had negative experiences may be underrepresented in our sample, potentially inflating acceptability and satisfaction scores. Fourth, the sample sizes for both components of this mixed-methods study were determined by program enrollment, survey completion rates, and staff availability rather than a priori sample size calculations. The small number of staff interviews limits the qualitative depth and generalizability of staff perspectives, and the survey sample size may have limited statistical power to detect smaller associations between participant characteristics and implementation outcomes in the regression analyses. Fifth, given the number of predictors tested across the regression models, some significant associations may reflect false positives; replication in larger samples is needed to confirm the associations identified here. Finally, brief free-response answers in the client surveys may have missed nuanced feedback. Future research should incorporate in-depth interviews with telePrEP clients to further elucidate their perspectives and experiences in participating in this model of PrEP care delivery.

## Conclusion

The Philadelphia TelePrEP Program demonstrates that telehealth-based PrEP delivery can achieve high user satisfaction when supported by dedicated navigation services, flexible hybrid care options, and strong organizational partnerships. However, realizing the full potential of telePrEP to advance health equity requires addressing persistent challenges including the digital divide, sustaining and expanding culturally responsive outreach strategies that extend beyond online platforms, and streamlining multi-organizational care coordination to reduce administrative burden on both staff and patients. As telePrEP programs continue to evolve, success will depend on balancing technological innovation with sustained investment in human-centered care, community partnerships, and operational infrastructure that can support equitable access to HIV prevention services.

## Supplementary Information

Below is the link to the electronic supplementary material.

Supplementary Material 1

## Data Availability

The datasets used in the current study are available from the corresponding author on reasonable request.

## Abbreviations

PrEP: Pre-exposure prophylaxis
PTP: Philadelphia TelePrEP Program
PDPH: Philadelphia Department of Public Health
JEPH: Jefferson Einstein Philadelphia Hospital
CFIR: Consolidated Framework for Implementation Research
