# Supplementary material for: Implementation determinants and outcomes in the Philadelphia TelePrEP Program: a mixed methods study of client and staff perspectives
Source: Implement Sci Commun. 2026 Jun 30;7:191. doi: 10.1186/s43058-026-01009-7 (PMC13595741; doi:10.1186/s43058-026-01009-7)
Supplement: Supplementary file 1 [file 43058_2026_1009_MOESM1_ESM.docx]

Supplemental Table 1. Checklist for Mixed Methods in Health Services Management Research

| # | Checklist Item | Location in Manuscript |
| --- | --- | --- |
| Rational and description of MMR design | | |
| 1 | 1. Provide a clear statement of the study purpose | Introduction (final paragraph) |
| 2 | Explicitly describe the MMR design in accordance with Creswell's (2015) typology and use a diagram to illustrate the relationship and sequence of qualitative and quantitative research components | Methods (Study Design section) – The concurrent design is described in text; visual integration is provided through Table 4 (Joint Display Table) |
| 3 | Justify why the MMR design is appropriate for meeting the study purpose | Methods (Study Design section) |
| Transparency in describing method details | | |
| 4 | Describe the study population(s) and sample(s; e.g., who, what, how many) | Methods (Interviews with Program Staff; Client Survey); Results (Participant Characteristics; Qualitative Results intro) |
| 5 | Describe the sampling procedures (including inclusion and exclusion criteria, recruitment) | Methods (Interviews with Program Staff; Client Survey) |
| 6 | Describe qualitative data collection processes (how often data were collected, who collected the data, what kind of data collection instruments were used, how data were recorded—e.g., notes, transcripts) | Methods (Interviews with Program Staff; Free Response Questions) |
| 7 | Describe quantitative data collection processes (how often data were collected, who collected the data, what kind of data collection instruments were used, measurements, validity/reliability) | Methods (Client Survey; Measures) |
| 8 | Describe qualitative data analysis processes (coding, single or multiple coders, replication logic, credibility) | Methods (Qualitative Analysis section: Interview and Survey Free-Response Data subsections) |
| 9 | Describe quantitative data analysis procedures (missing data and how they are handled, statistical tests used) | Methods (Quantitative Analysis section) |
| Integration of qualitative and quantitative research components | | |
| 10 | Interpret qualitative analysis results with appropriate quotes if necessary | Results (Qualitative Results and Mixed Methods Integration section, Themes 1-7, each with supporting quotes) |
| 11 | Interpret quantitative analysis results in consideration of statistical significance, selection bias, and threats to validity | Discussion (Limitations section) |
| 12 | Compare qualitative and quantitative results | Results (Table 4 Joint Display Table) |
| 13 | Address divergencies and inconsistencies between qualitative and quantitative results | Results (Table 4 Joint Display Table) |
